# Poly[[dodeca­aqua­bis­(μ_3_-pyridine-2,6-dicarboxyl­ato)tetra­kis­(μ_2_-pyridine-2,6-dicarboxyl­ato)tri­calciumdieuropium(III)] 10.5-hydrate]

**DOI:** 10.1107/S1600536812018028

**Published:** 2012-04-28

**Authors:** Fengjuan Shi, Jiguang Deng, Hongxing Dai

**Affiliations:** a Laboratory of Catalysis Chemistry and Nanoscience, Department of Chemistry and Chemical Engineering, College of Environmental and Energy Engineering, Beijing University of Technology, Beijing 100124, People’s Republic of China

## Abstract

In the title compound, {[Ca_3_Eu_2_(C_7_H_3_NO_4_)_6_(H_2_O)_12_]·10.5H_2_O}_*n*_, the Eu^III^ ion is nine-coordinated by three tridentate pyridine-2,6-dicarboxyl­ate (PDA) ligands, forming a [Eu(PDA)_3_]^3−^ building block. The Ca^2+^ ions adopt two types of coordination geometries. One Ca^2+^ ion, lying on a twofold rotation axis, is eight-coordinated by four carboxyl­ate O atoms from four PDA ligands and four water mol­ecules, and the other two Ca^2+^ ions, each lying on an inversion center, are six-coordinated by two carboxyl­ate O atoms from two PDA ligands and four water mol­ecules. The carboxyl­ate groups bridge the Eu^III^ and Ca^2+^ ions into a three-dimensional porous framework, with channels extending along [010] and [001] in which lattice water mol­ecules are located. Two of the lattice water mol­ecules are disordered over two sets of sites with equal occupancy and one water mol­ecule is 0.25-occupied. Numerous O—H⋯O hydrogen bonds involving the water mol­ecules and carboxyl­ate O atoms are present.

## Related literature

For 3*d*–4*f* and 4*d*–4*f* metal complexes with pyridine-2,6-dicarboxyl­ate ligands, see: Zhao *et al.* (2006 ▶, 2007 ▶, 2011 ▶); Zhao, Zhao *et al.* (2009 ▶). For *Ln*–Ba (*Ln* = lanthanide) complexes with pyridine-2,6-dicarboxyl­ate ligands, see: Zhao, Zuo *et al.* (2009 ▶).
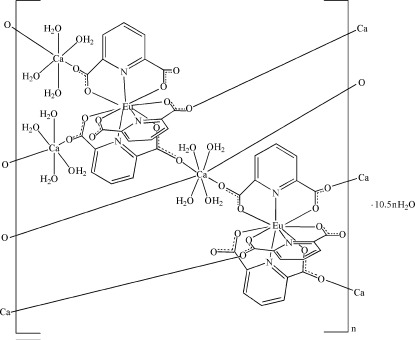


## Experimental

### 

#### Crystal data

[Ca_3_Eu_2_(C_7_H_3_NO_4_)_6_(H_2_O)_12_]·10.5H_2_O
*M* *_r_* = 1820.14
Monoclinic, 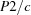

*a* = 16.070 (4) Å
*b* = 9.471 (2) Å
*c* = 23.540 (6) Å
β = 107.685 (4)°
*V* = 3413.5 (14) Å^3^
*Z* = 2
Mo *K*α radiation
μ = 2.16 mm^−1^
*T* = 113 K
0.20 × 0.19 × 0.16 mm

#### Data collection

Rigaku Saturn724 CCD diffractometer
Absorption correction: multi-scan (*CrystalClear*; Rigaku/MSC, 2009 ▶) *T*_min_ = 0.672, *T*_max_ = 0.724
27531 measured reflections
6015 independent reflections
4964 reflections with *I* > 2σ(*I*)
*R*_int_ = 0.054

#### Refinement

*R*[*F* ^2^ > 2σ(*F* ^2^)] = 0.042
*wR*(*F* ^2^) = 0.094
*S* = 1.14
6015 reflections
531 parameters
30 restraints
H atoms treated by a mixture of independent and constrained refinement
Δρ_max_ = 1.31 e Å^−3^
Δρ_min_ = −1.29 e Å^−3^

### 

Data collection: *CrystalClear* (Rigaku/MSC, 2009 ▶); cell refinement: *CrystalClear*; data reduction: *CrystalClear*; program(s) used to solve structure: *SHELXS97* (Sheldrick, 2008 ▶); program(s) used to refine structure: *SHELXL97* (Sheldrick, 2008 ▶); molecular graphics: *XP* in *SHELXTL* (Sheldrick, 2008 ▶); software used to prepare material for publication: *SHELXTL*.

## Supplementary Material

Crystal structure: contains datablock(s) global, I. DOI: 10.1107/S1600536812018028/hy2534sup1.cif

Structure factors: contains datablock(s) I. DOI: 10.1107/S1600536812018028/hy2534Isup2.hkl

Additional supplementary materials:  crystallographic information; 3D view; checkCIF report

## Figures and Tables

**Table 1:** Hydrogen-bond geometry (Å, °)

*D*—H⋯*A*	*D*—H	H⋯*A*	*D*⋯*A*	*D*—H⋯*A*
O13—H13*A*⋯O10^i^	0.85 (1)	1.97 (2)	2.796 (6)	163 (5)
O13—H13*B*⋯O20^ii^	0.85 (1)	1.85 (1)	2.696 (6)	175 (6)
O14—H14*A*⋯O16^iii^	0.85 (1)	1.96 (2)	2.774 (6)	160 (5)
O14—H14*B*⋯O9^iii^	0.85 (1)	1.91 (2)	2.723 (5)	160 (5)
O15—H15*A*⋯O21^iv^	0.85 (1)	1.99 (1)	2.839 (6)	172 (6)
O15—H15*B*⋯O5	0.85 (1)	1.90 (1)	2.734 (5)	165 (5)
O16—H16*A*⋯O13^iv^	0.78 (6)	2.38 (7)	3.079 (6)	150 (6)
O16—H16*B*⋯O1^iv^	0.84 (6)	1.88 (7)	2.704 (6)	169 (6)
O17—H17*A*⋯O3^iii^	0.85 (1)	1.91 (1)	2.745 (6)	167 (5)
O17—H17*B*⋯O19^v^	0.85 (1)	1.93 (3)	2.722 (7)	155 (6)
O18—H18*A*⋯O22′^vi^	0.85 (1)	2.37 (6)	2.990 (15)	130 (6)
O18—H18*A*⋯O22^vi^	0.85 (1)	2.16 (7)	2.657 (14)	117 (6)
O18—H18*B*⋯O4^iii^	0.85 (1)	1.98 (2)	2.772 (6)	155 (5)
O19—H19*A*⋯O24^vii^	0.86 (1)	2.31 (3)	3.12 (2)	159 (6)
O19—H19*B*⋯O7^viii^	0.85 (1)	2.07 (4)	2.828 (6)	148 (6)
O20—H20*A*⋯O15	0.85 (1)	2.04 (2)	2.862 (6)	161 (6)
O20—H20*B*⋯O23	0.85 (1)	2.03 (4)	2.758 (10)	143 (6)
O20—H20*B*⋯O22′^ix^	0.85 (1)	2.32 (4)	3.042 (13)	143 (5)
O21—H21*A*⋯O11	0.85 (1)	1.94 (2)	2.774 (6)	167 (6)
O21—H21*B*⋯O17	0.85 (1)	2.42 (4)	3.113 (7)	138 (5)

Symmetry codes: (i) 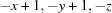; (ii) 
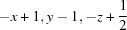
; (iii) 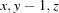; (iv) 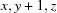; (v) 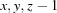; (vi) 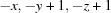; (vii) 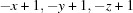; (viii) 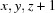; (ix) 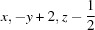.

**Fig. 1.**
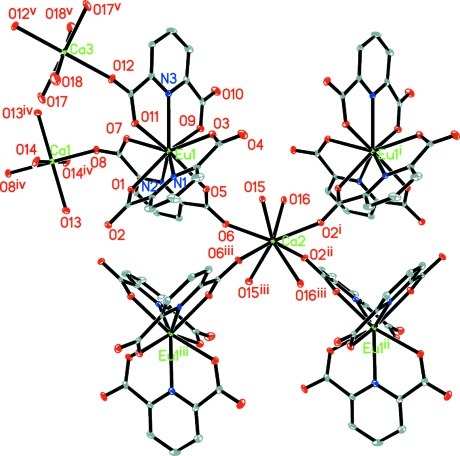
The asymmetric unit of the title compound. Displacement ellipsoids are drawn at the 30% probability level. Lattice water molecules and H atoms are omitted and C atoms are not labeled for clarity. [Symmetry codes: (i) x, y+1, z; (ii) -x+1, y+1, -z+1/2; (iii) -x+1, y, -z+1/2; (iv) -x+1, -y, -z; (v) -x, -y, -z.]

**Fig. 2.**
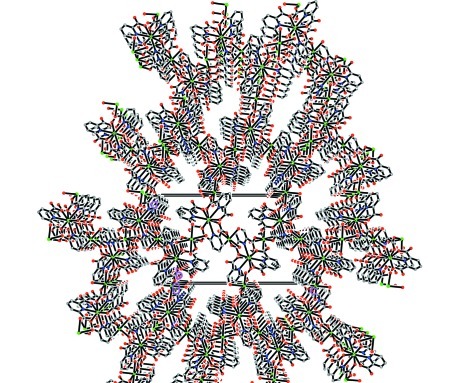
The three-dimensional structure of the title compound viewed along the b axis. Lattice water molecules and H atoms are omitted for clarity.

## supplementary crystallographic information

### Comment

Pyridine-2,6-dicarboxylic acid (H_2_PDA), as a chelating or bridging ligand, has been proven to be an efficient multidentate ligand to construct multidimensional heterometal-organic frameworks with porous structures. Especially, the lanthanide complexes based on H_2_PDA show variable structure characters. The PDA anion chelating to a lanthanide ion possesses free carboxylate oxygen atoms, which can coordinate with transition metals. A systematic study of 3d–4f and 4d–4f complexes based on pyridine-2,6-dicarboxylic acid ligand has been undertaken, such as Ln–Mn (Zhao *et al.*, 2006), Ln–Co (Zhao *et al.*, 2007), Ln–Fe (Zhao *et al.*, 2011) and Ln–Ag (Zhao, Zhao *et al.*, 2009). However, the reports of heterometal-organic frameworks associated with lanthanide and alkaline earth ions are rather rare, only Ln–Ba complexes based on pyridine-2,6-dicarboxylic acid ligand were reported (Zhao, Zuo *et al.*, 2009). In this paper, we report the synthesis and crystal structure of the title compound, using the hydrothermal method with pyridine-2,6-dicarboxylic acid.

In the title compound, the Eu^III^ ion coordinates with three PDA ligands in a tridentate mode, forming a [Eu(PDA)_3_]^3-^ building block. The remaining coordination sites in the [Eu(PDA)_3_]^3-^ unit coordinate with Ca^2+^ ions. The Ca^2+^ ions adopt two types of coordination geometry, as shown in Fig. 1. The coordination environments of Ca1 and Ca3 atoms are similar, each located on an inversion center and coordinated by two carboxylate O atoms and four water molecules, forming [CaO_2_(H_2_O)_4_] building blocks. Ca2 atom lies on a twofold rotation axis and is coordinated by four carboxylate O atoms from four PDA ligands and four water molecules, forming an eight-coordinated [CaO_4_(H_2_O)_4_] building block. Furthermore, Ca2 and Ca1 atoms are bridged by the same PDA ligand. The overall framework can be viewed as the self-assembly of three types of building blocks, [Eu(PDA)_3_], [CaO_2_(H_2_O)_4_] and [CaO_4_(H_2_O)_4_]. Each [Eu(PDA)_3_] is surrounded by two [CaO_2_(H_2_O)_4_] units and one [CaO_4_(H_2_O)_4_] unit in its vicinity, while each [CaO_2_(H_2_O)_4_] or [CaO_4_(H_2_O)_4_] unit has two [Eu(PDA)_3_] and four [Eu(PDA)_3_] units as the nearest neighbors. The linkers are carboxylate groups. As a result of this connection, the [CaO_2_(H_2_O)_4_], [CaO_4_(H_2_O)_4_] and [Eu(PDA)_3_] building blocks are connected alternately by carboxylate groups, forming a three-dimensional porous framework, with channels extending along [0 1 0] and [0 0 1] (Fig. 2).

### Experimental

A mixture of pyridine-2,6-dicarboxylic acid (134 mg, 0.8 mmol), calcium hydroxide (52 mg, 0.7 mmol), europium nitrate hexahydrate (89 mg, 0.2 mmol) and deionized water (10 ml) was placed in a 25 ml Teflon-lined stainless steel autoclave, which was kept at 433 K for 3 days. The resuling colorless block-shaped crystals suitable for X-ray diffraction experiment were collected and washed with deionized water and diethyl ether (yield: 170 mg).

### Refinement

H atoms bonded to C atoms were positioned geometrically and refined using a riding model, with C—H = 0.95 Å and *U*_iso_(H) = 1.2*U*_eq_(C). H atorms of water molecules were found from difference Fourier maps and refined with a distance restraint of O—H = 0.85 (1) Å and with *U*_iso_(H) = 1.2*U*_eq_(O). O22 and O23 atoms were disordered over two sets of positions with occupancy factors of 0.5:0.5, respectively. The occupancy factor of the lattice water O24 was set to 0.25. H atorms of disordered water molecules O22, O23 and O24 were not included. The highest residual electron density was found 2.36 Å from H5 atom and the deepest hole 0.78 Å from Eu1 atom.

### Crystal data

**Table:**

[Ca_3_Eu_2_(C_7_H_3_NO_4_)_6_(H_2_O)_12_]·10.5H_2_O	*F*(000) = 1830
*M**_r_* = 1820.14	*D*_x_ = 1.771 Mg m^−^^3^
Monoclinic, *P*2/*c*	Mo *K*α radiation, λ = 0.71073 Å
Hall symbol: -P 2yc	Cell parameters from 11834 reflections
*a* = 16.070 (4) Å	θ = 1.3–27.9°
*b* = 9.471 (2) Å	µ = 2.16 mm^−^^1^
*c* = 23.540 (6) Å	*T* = 113 K
β = 107.685 (4)°	Prism, colorless
*V* = 3413.5 (14) Å^3^	0.20 × 0.19 × 0.16 mm
*Z* = 2	

### Data collection

**Table:**

Rigaku Saturn724 CCD diffractometer	6015 independent reflections
Radiation source: rotating anode	4964 reflections with *I* > 2σ(*I*)
Multilayer monochromator	*R*_int_ = 0.054
Detector resolution: 14.22 pixels mm^-1^	θ_max_ = 25.0°, θ_min_ = 1.3°
ω and φ scans	*h* = −18→19
Absorption correction: multi-scan (*CrystalClear*; Rigaku/MSC, 2009)	*k* = −10→11
*T*_min_ = 0.672, *T*_max_ = 0.724	*l* = −28→28
27531 measured reflections	

### Refinement

**Table:**

Refinement on *F*^2^	Primary atom site location: structure-invariant direct methods
Least-squares matrix: full	Secondary atom site location: difference Fourier map
*R*[*F*^2^ > 2σ(*F*^2^)] = 0.042	Hydrogen site location: inferred from neighbouring sites
*wR*(*F*^2^) = 0.094	H atoms treated by a mixture of independent and constrained refinement
*S* = 1.14	*w* = 1/[σ^2^(*F*_o_^2^) + (0.0212*P*)^2^ + 16.8621*P*] where *P* = (*F*_o_^2^ + 2*F*_c_^2^)/3
6015 reflections	(Δ/σ)_max_ = 0.002
531 parameters	Δρ_max_ = 1.31 e Å^−^^3^
30 restraints	Δρ_min_ = −1.29 e Å^−^^3^

### Special details

**Table:**

Geometry. All e.s.d.'s (except the e.s.d. in the dihedral angle between two l.s. planes) are estimated using the full covariance matrix. The cell e.s.d.'s are taken into account individually in the estimation of e.s.d.'s in distances, angles and torsion angles; correlations between e.s.d.'s in cell parameters are only used when they are defined by crystal symmetry. An approximate (isotropic) treatment of cell e.s.d.'s is used for estimating e.s.d.'s involving l.s. planes.
Refinement. Refinement of *F*^2^ against ALL reflections. The weighted *R*-factor *wR* and goodness of fit *S* are based on *F*^2^, conventional *R*-factors *R* are based on *F*, with *F* set to zero for negative *F*^2^. The threshold expression of *F*^2^ > σ(*F*^2^) is used only for calculating *R*-factors(gt) *etc*. and is not relevant to the choice of reflections for refinement. *R*-factors based on *F*^2^ are statistically about twice as large as those based on *F*, and *R*- factors based on ALL data will be even larger.

### Fractional atomic coordinates and isotropic or equivalent isotropic displacement parameters (Å^2^)

**Table:**

	*x*	*y*	*z*	*U*_iso_*/*U*_eq_	Occ. (<1)
Eu1	0.296251 (16)	0.50554 (3)	0.097315 (11)	0.00952 (9)	
Ca1	0.5000	0.0000	0.0000	0.0113 (3)	
Ca2	0.5000	1.00350 (17)	0.2500	0.0138 (3)	
Ca3	0.0000	0.0000	0.0000	0.0198 (4)	
O1	0.3736 (2)	0.3233 (4)	0.16621 (16)	0.0151 (8)	
O2	0.4013 (3)	0.2000 (4)	0.25127 (16)	0.0182 (9)	
O3	0.1929 (2)	0.6789 (4)	0.11297 (16)	0.0174 (9)	
O4	0.1130 (3)	0.7722 (5)	0.16683 (18)	0.0282 (11)	
O5	0.3886 (2)	0.6872 (4)	0.15705 (16)	0.0149 (8)	
O6	0.5155 (2)	0.8058 (4)	0.18919 (16)	0.0158 (9)	
O7	0.3344 (2)	0.3313 (4)	0.03339 (16)	0.0140 (8)	
O8	0.4335 (2)	0.2095 (4)	0.00428 (16)	0.0169 (9)	
O9	0.2999 (2)	0.6871 (4)	0.02212 (16)	0.0162 (9)	
O10	0.2495 (3)	0.7858 (4)	−0.06830 (18)	0.0243 (10)	
O11	0.1823 (2)	0.3273 (4)	0.08688 (16)	0.0159 (9)	
O12	0.0604 (3)	0.2142 (4)	0.03527 (18)	0.0205 (9)	
O13	0.6116 (2)	0.0662 (4)	0.08852 (17)	0.0185 (9)	
H13A	0.647 (2)	0.113 (5)	0.075 (2)	0.022*	
H13B	0.639 (3)	0.012 (5)	0.1167 (18)	0.022*	
O14	0.4145 (3)	−0.0939 (4)	0.05348 (17)	0.0222 (10)	
H14A	0.419 (4)	−0.043 (5)	0.0840 (16)	0.027*	
H14B	0.380 (3)	−0.162 (4)	0.052 (2)	0.027*	
O15	0.3478 (3)	0.9194 (4)	0.21233 (17)	0.0204 (9)	
H15A	0.306 (3)	0.974 (5)	0.194 (2)	0.025*	
H15B	0.354 (4)	0.854 (4)	0.1894 (18)	0.025*	
O16	0.4648 (3)	1.0983 (4)	0.14661 (18)	0.0165 (9)	
H16A	0.504 (4)	1.121 (7)	0.135 (3)	0.020*	
H16B	0.437 (4)	1.172 (7)	0.148 (3)	0.020*	
O17	0.1405 (3)	−0.0914 (5)	0.0396 (2)	0.0314 (11)	
H17A	0.164 (3)	−0.161 (4)	0.061 (3)	0.038*	
H17B	0.179 (3)	−0.033 (5)	0.036 (3)	0.038*	
O18	−0.0140 (3)	−0.0554 (5)	0.0933 (2)	0.0391 (12)	
H18A	−0.0678 (13)	−0.048 (7)	0.090 (3)	0.047*	
H18B	0.011 (3)	−0.118 (6)	0.118 (3)	0.047*	
N1	0.2624 (3)	0.4885 (5)	0.19488 (18)	0.0125 (9)	
N2	0.4526 (3)	0.5088 (4)	0.09633 (18)	0.0106 (9)	
N3	0.1693 (3)	0.5085 (5)	0.00041 (19)	0.0132 (9)	
C1	0.3627 (4)	0.2941 (6)	0.2164 (2)	0.0140 (12)	
C2	0.2960 (4)	0.3823 (6)	0.2330 (2)	0.0158 (12)	
C3	0.2680 (4)	0.3533 (7)	0.2820 (2)	0.0227 (14)	
H3	0.2916	0.2765	0.3078	0.027*	
C4	0.2045 (4)	0.4396 (8)	0.2922 (3)	0.0292 (16)	
H4	0.1838	0.4223	0.3253	0.035*	
C5	0.1711 (4)	0.5518 (7)	0.2539 (3)	0.0281 (16)	
H5	0.1283	0.6128	0.2608	0.034*	
C6	0.2015 (4)	0.5727 (6)	0.2057 (2)	0.0184 (13)	
C7	0.1659 (4)	0.6849 (6)	0.1585 (2)	0.0170 (13)	
C8	0.4686 (4)	0.7088 (6)	0.1610 (2)	0.0124 (12)	
C9	0.5084 (3)	0.6070 (5)	0.1267 (2)	0.0119 (12)	
C10	0.5932 (4)	0.6158 (6)	0.1249 (2)	0.0169 (12)	
H10	0.6311	0.6876	0.1464	0.020*	
C11	0.6218 (4)	0.5190 (6)	0.0915 (2)	0.0182 (13)	
H11	0.6800	0.5233	0.0896	0.022*	
C12	0.5646 (4)	0.4142 (6)	0.0603 (2)	0.0165 (12)	
H12	0.5832	0.3458	0.0372	0.020*	
C13	0.4797 (3)	0.4130 (5)	0.0640 (2)	0.0116 (12)	
C14	0.4114 (4)	0.3098 (6)	0.0316 (2)	0.0134 (12)	
C15	0.2437 (4)	0.7017 (6)	−0.0285 (3)	0.0172 (13)	
C16	0.1646 (4)	0.6081 (6)	−0.0416 (2)	0.0147 (12)	
C17	0.0927 (4)	0.6196 (6)	−0.0923 (2)	0.0192 (13)	
H17	0.0898	0.6920	−0.1207	0.023*	
C18	0.0254 (4)	0.5236 (7)	−0.1002 (3)	0.0258 (15)	
H18	−0.0243	0.5285	−0.1346	0.031*	
C19	0.0312 (4)	0.4197 (6)	−0.0574 (3)	0.0207 (14)	
H19	−0.0142	0.3524	−0.0622	0.025*	
C20	0.1038 (3)	0.4157 (6)	−0.0079 (2)	0.0137 (12)	
C21	0.1156 (4)	0.3100 (6)	0.0415 (2)	0.0149 (12)	
O19	0.2379 (3)	0.0820 (5)	0.9928 (2)	0.0386 (12)	
H19A	0.211 (4)	0.121 (6)	0.9594 (17)	0.046*	
H19B	0.282 (3)	0.132 (6)	1.011 (2)	0.046*	
O20	0.2972 (3)	0.9062 (5)	0.3187 (2)	0.0351 (12)	
H20A	0.314 (4)	0.890 (7)	0.2883 (16)	0.042*	
H20B	0.253 (3)	0.960 (6)	0.309 (2)	0.042*	
O21	0.1986 (3)	0.0862 (5)	0.15634 (18)	0.0290 (11)	
H21A	0.196 (4)	0.152 (4)	0.1311 (18)	0.035*	
H21B	0.176 (4)	0.011 (3)	0.139 (2)	0.035*	
O22	0.1015 (10)	0.9136 (14)	0.8459 (6)	0.049 (4)	0.50
O23	0.1373 (5)	0.9971 (10)	0.2459 (3)	0.030 (2)	0.50
O22'	0.1367 (9)	0.9633 (14)	0.8406 (7)	0.049 (4)	0.50
O23'	0.0826 (8)	1.1594 (14)	0.2299 (5)	0.066 (4)	0.50
O24	0.8088 (12)	0.744 (2)	0.1251 (9)	0.039 (5)	0.25

### Atomic displacement parameters (Å^2^)

**Table:**

	*U*^11^	*U*^22^	*U*^33^	*U*^12^	*U*^13^	*U*^23^
Eu1	0.00769 (14)	0.00855 (14)	0.01199 (14)	−0.00043 (12)	0.00250 (10)	−0.00100 (12)
Ca1	0.0110 (7)	0.0095 (7)	0.0141 (7)	0.0012 (7)	0.0049 (6)	−0.0014 (7)
Ca2	0.0153 (8)	0.0103 (8)	0.0120 (7)	0.000	−0.0018 (6)	0.000
Ca3	0.0147 (8)	0.0103 (8)	0.0264 (9)	−0.0014 (7)	−0.0059 (7)	−0.0002 (7)
O1	0.015 (2)	0.014 (2)	0.016 (2)	0.0042 (16)	0.0041 (16)	−0.0006 (16)
O2	0.021 (2)	0.014 (2)	0.015 (2)	0.0040 (17)	−0.0005 (17)	0.0014 (17)
O3	0.014 (2)	0.018 (2)	0.020 (2)	0.0036 (17)	0.0044 (17)	−0.0012 (17)
O4	0.023 (2)	0.036 (3)	0.025 (2)	0.016 (2)	0.0068 (19)	−0.003 (2)
O5	0.009 (2)	0.014 (2)	0.021 (2)	−0.0012 (16)	0.0038 (16)	−0.0036 (16)
O6	0.010 (2)	0.013 (2)	0.022 (2)	−0.0035 (16)	0.0008 (16)	−0.0036 (17)
O7	0.012 (2)	0.013 (2)	0.018 (2)	−0.0009 (16)	0.0071 (16)	−0.0035 (16)
O8	0.023 (2)	0.013 (2)	0.017 (2)	0.0045 (17)	0.0090 (17)	−0.0017 (16)
O9	0.014 (2)	0.016 (2)	0.018 (2)	0.0002 (17)	0.0050 (17)	0.0032 (16)
O10	0.021 (2)	0.026 (2)	0.027 (2)	0.0012 (19)	0.0096 (19)	0.0131 (19)
O11	0.010 (2)	0.015 (2)	0.020 (2)	−0.0034 (16)	0.0012 (17)	−0.0025 (16)
O12	0.015 (2)	0.013 (2)	0.032 (2)	−0.0064 (17)	0.0043 (18)	−0.0052 (17)
O13	0.015 (2)	0.021 (2)	0.018 (2)	−0.0004 (18)	0.0024 (17)	0.0034 (17)
O14	0.033 (3)	0.018 (2)	0.019 (2)	−0.0128 (19)	0.014 (2)	−0.0057 (17)
O15	0.019 (2)	0.013 (2)	0.023 (2)	0.0057 (18)	−0.0025 (18)	−0.0045 (17)
O16	0.019 (2)	0.010 (2)	0.020 (2)	0.0020 (18)	0.0057 (18)	−0.0018 (17)
O17	0.015 (2)	0.016 (2)	0.061 (3)	0.0024 (18)	0.007 (2)	0.014 (2)
O18	0.042 (3)	0.028 (3)	0.046 (3)	0.007 (2)	0.012 (3)	0.005 (2)
N1	0.010 (2)	0.013 (2)	0.014 (2)	−0.001 (2)	0.0023 (18)	−0.005 (2)
N2	0.012 (2)	0.006 (2)	0.014 (2)	−0.001 (2)	0.0042 (18)	0.0029 (19)
N3	0.011 (2)	0.013 (2)	0.017 (2)	0.000 (2)	0.0055 (18)	−0.004 (2)
C1	0.017 (3)	0.012 (3)	0.011 (3)	−0.008 (2)	0.001 (2)	−0.003 (2)
C2	0.011 (3)	0.018 (3)	0.015 (3)	−0.003 (2)	−0.002 (2)	−0.006 (2)
C3	0.019 (3)	0.034 (4)	0.014 (3)	−0.003 (3)	0.003 (3)	0.003 (3)
C4	0.017 (3)	0.052 (4)	0.019 (3)	0.004 (3)	0.008 (3)	0.005 (3)
C5	0.016 (3)	0.052 (4)	0.019 (3)	0.001 (3)	0.009 (3)	−0.010 (3)
C6	0.009 (3)	0.023 (3)	0.019 (3)	−0.001 (2)	−0.003 (2)	−0.007 (2)
C7	0.010 (3)	0.022 (3)	0.017 (3)	0.001 (3)	0.001 (2)	−0.007 (2)
C8	0.014 (3)	0.010 (3)	0.011 (3)	0.000 (2)	0.001 (2)	0.002 (2)
C9	0.010 (3)	0.009 (3)	0.016 (3)	0.002 (2)	0.002 (2)	0.005 (2)
C10	0.013 (3)	0.018 (3)	0.020 (3)	−0.001 (2)	0.004 (2)	0.005 (2)
C11	0.012 (3)	0.020 (3)	0.022 (3)	0.005 (2)	0.005 (2)	0.008 (2)
C12	0.015 (3)	0.015 (3)	0.021 (3)	0.004 (2)	0.009 (2)	0.003 (2)
C13	0.012 (3)	0.008 (3)	0.015 (3)	0.004 (2)	0.005 (2)	0.006 (2)
C14	0.017 (3)	0.010 (3)	0.013 (3)	0.002 (2)	0.006 (2)	0.005 (2)
C15	0.020 (3)	0.012 (3)	0.023 (3)	0.007 (2)	0.010 (3)	0.001 (2)
C16	0.015 (3)	0.014 (3)	0.019 (3)	0.006 (2)	0.012 (2)	0.001 (2)
C17	0.015 (3)	0.025 (3)	0.020 (3)	0.004 (3)	0.008 (3)	0.003 (3)
C18	0.014 (3)	0.039 (4)	0.020 (3)	0.007 (3)	−0.002 (2)	−0.002 (3)
C19	0.013 (3)	0.018 (3)	0.025 (3)	0.000 (3)	−0.002 (3)	−0.009 (3)
C20	0.011 (3)	0.013 (3)	0.019 (3)	0.000 (2)	0.006 (2)	−0.006 (2)
C21	0.013 (3)	0.010 (3)	0.023 (3)	0.000 (2)	0.008 (3)	−0.004 (2)
O19	0.032 (3)	0.038 (3)	0.048 (3)	−0.013 (2)	0.014 (2)	−0.013 (2)
O20	0.038 (3)	0.035 (3)	0.031 (3)	0.010 (2)	0.008 (2)	−0.011 (2)
O21	0.029 (3)	0.016 (2)	0.031 (3)	−0.002 (2)	−0.007 (2)	0.0055 (19)
O22	0.070 (11)	0.046 (9)	0.048 (7)	0.012 (7)	0.041 (7)	0.017 (6)
O23	0.023 (5)	0.048 (6)	0.017 (4)	−0.007 (5)	0.003 (4)	−0.005 (4)
O22'	0.040 (8)	0.041 (8)	0.071 (9)	−0.001 (6)	0.025 (6)	0.026 (6)
O23'	0.068 (8)	0.085 (10)	0.045 (7)	−0.036 (7)	0.018 (6)	−0.015 (6)
O24	0.034 (6)	0.040 (6)	0.043 (6)	0.003 (5)	0.013 (5)	0.003 (5)

### Geometric parameters (Å, º)

**Table:**

Eu1—O5	2.423 (4)	O14—H14A	0.85 (1)
Eu1—O7	2.434 (4)	O14—H14B	0.85 (1)
Eu1—O1	2.435 (4)	O15—H15A	0.85 (1)
Eu1—O3	2.441 (4)	O15—H15B	0.85 (1)
Eu1—O11	2.447 (4)	O16—H16A	0.78 (6)
Eu1—O9	2.481 (4)	O16—H16B	0.84 (6)
Eu1—N2	2.520 (4)	O17—H17A	0.85 (1)
Eu1—N1	2.521 (4)	O17—H17B	0.85 (1)
Eu1—N3	2.557 (4)	O18—H18A	0.85 (1)
Ca1—O8^i^	2.270 (4)	O18—H18B	0.85 (1)
Ca1—O8	2.270 (4)	N1—C6	1.345 (7)
Ca1—O14^i^	2.305 (4)	N1—C2	1.347 (7)
Ca1—O14	2.305 (4)	N2—C9	1.338 (7)
Ca1—O13^i^	2.383 (4)	N2—C13	1.340 (7)
Ca1—O13	2.383 (4)	N3—C20	1.339 (7)
Ca1—H13A	2.70 (5)	N3—C16	1.353 (7)
Ca1—H14A	2.70 (4)	C1—C2	1.501 (8)
Ca2—O6^ii^	2.415 (4)	C2—C3	1.387 (8)
Ca2—O6	2.415 (4)	C3—C4	1.383 (9)
Ca2—O2^iii^	2.451 (4)	C3—H3	0.9500
Ca2—O2^iv^	2.451 (4)	C4—C5	1.391 (9)
Ca2—O15^ii^	2.466 (4)	C4—H4	0.9500
Ca2—O15	2.466 (4)	C5—C6	1.379 (8)
Ca2—O16^ii^	2.493 (4)	C5—H5	0.9500
Ca2—O16	2.493 (4)	C6—C7	1.519 (8)
Ca2—H15B	2.74 (5)	C8—C9	1.519 (7)
Ca3—O12	2.292 (4)	C9—C10	1.378 (8)
Ca3—O12^v^	2.292 (4)	C10—C11	1.375 (8)
Ca3—O17^v^	2.332 (4)	C10—H10	0.9500
Ca3—O17	2.332 (4)	C11—C12	1.400 (8)
Ca3—O18	2.334 (5)	C11—H11	0.9500
Ca3—O18^v^	2.334 (5)	C12—C13	1.392 (8)
Ca3—H17B	2.76 (4)	C12—H12	0.9500
Ca3—H18A	2.70 (5)	C13—C14	1.495 (8)
O1—C1	1.276 (6)	C15—C16	1.502 (8)
O2—C1	1.243 (6)	C16—C17	1.390 (8)
O2—Ca2^vi^	2.451 (4)	C17—C18	1.381 (8)
O3—C7	1.274 (7)	C17—H17	0.9500
O4—C7	1.243 (7)	C18—C19	1.392 (9)
O5—C8	1.277 (6)	C18—H18	0.9500
O6—C8	1.244 (6)	C19—C20	1.375 (8)
O7—C14	1.267 (6)	C19—H19	0.9500
O8—C14	1.257 (6)	C20—C21	1.501 (8)
O9—C15	1.266 (7)	O19—H19A	0.86 (1)
O10—C15	1.254 (7)	O19—H19B	0.85 (1)
O11—C21	1.274 (6)	O20—H20A	0.85 (1)
O12—C21	1.247 (7)	O20—H20B	0.85 (1)
O13—H13A	0.85 (1)	O21—H21A	0.85 (1)
O13—H13B	0.85 (1)	O21—H21B	0.85 (1)
			
O5—Eu1—O7	127.97 (12)	O12^v^—Ca3—H17B	107.2 (11)
O5—Eu1—O1	91.03 (12)	O17^v^—Ca3—H17B	163.3 (7)
O7—Eu1—O1	75.97 (12)	O17—Ca3—H17B	16.7 (7)
O5—Eu1—O3	76.07 (12)	O18—Ca3—H17B	94.3 (12)
O7—Eu1—O3	149.31 (12)	O18^v^—Ca3—H17B	85.7 (12)
O1—Eu1—O3	127.54 (13)	O12—Ca3—H18A	95.3 (15)
O5—Eu1—O11	148.83 (13)	O12^v^—Ca3—H18A	84.7 (15)
O7—Eu1—O11	77.98 (12)	O17^v^—Ca3—H18A	78.7 (7)
O1—Eu1—O11	78.07 (12)	O17—Ca3—H18A	101.3 (7)
O3—Eu1—O11	87.52 (13)	O18—Ca3—H18A	17.5 (8)
O5—Eu1—O9	77.22 (12)	O18^v^—Ca3—H18A	162.5 (8)
O7—Eu1—O9	87.93 (12)	H17B—Ca3—H18A	111.7 (14)
O1—Eu1—O9	148.04 (13)	C1—O1—Eu1	125.7 (3)
O3—Eu1—O9	78.80 (13)	C1—O2—Ca2^vi^	137.0 (4)
O11—Eu1—O9	125.83 (12)	C7—O3—Eu1	125.3 (3)
O5—Eu1—N2	63.91 (13)	C8—O5—Eu1	125.6 (3)
O7—Eu1—N2	64.10 (13)	C8—O6—Ca2	137.2 (4)
O1—Eu1—N2	72.89 (13)	C14—O7—Eu1	124.2 (3)
O3—Eu1—N2	135.96 (13)	C14—O8—Ca1	153.3 (3)
O11—Eu1—N2	136.48 (13)	C15—O9—Eu1	125.7 (4)
O9—Eu1—N2	75.26 (13)	C21—O11—Eu1	125.9 (3)
O5—Eu1—N1	77.31 (13)	C21—O12—Ca3	153.5 (4)
O7—Eu1—N1	133.20 (13)	Ca1—O13—H13A	103 (4)
O1—Eu1—N1	63.64 (13)	Ca1—O13—H13B	127 (4)
O3—Eu1—N1	63.93 (13)	H13A—O13—H13B	111 (4)
O11—Eu1—N1	71.66 (13)	Ca1—O14—H14A	109 (3)
O9—Eu1—N1	138.80 (13)	Ca1—O14—H14B	141 (3)
N2—Eu1—N1	120.06 (13)	H14A—O14—H14B	111 (5)
O5—Eu1—N3	133.78 (14)	Ca2—O15—H15A	122 (4)
O7—Eu1—N3	74.84 (13)	Ca2—O15—H15B	99 (4)
O1—Eu1—N3	135.19 (13)	H15A—O15—H15B	110 (5)
O3—Eu1—N3	74.49 (13)	Ca2—O16—H16A	118 (5)
O11—Eu1—N3	63.13 (13)	Ca2—O16—H16B	103 (4)
O9—Eu1—N3	62.70 (13)	H16A—O16—H16B	106 (6)
N2—Eu1—N3	121.28 (13)	Ca3—O17—H17A	137 (4)
N1—Eu1—N3	118.61 (14)	Ca3—O17—H17B	111 (4)
O8^i^—Ca1—O8	179.999 (2)	H17A—O17—H17B	111 (5)
O8^i^—Ca1—O14^i^	86.94 (14)	Ca3—O18—H18A	107 (4)
O8—Ca1—O14^i^	93.06 (14)	Ca3—O18—H18B	131 (5)
O8^i^—Ca1—O14	93.07 (14)	H18A—O18—H18B	112 (6)
O8—Ca1—O14	86.93 (14)	C6—N1—C2	119.0 (5)
O14^i^—Ca1—O14	180.0	C6—N1—Eu1	120.3 (4)
O8^i^—Ca1—O13^i^	88.30 (14)	C2—N1—Eu1	120.0 (4)
O8—Ca1—O13^i^	91.70 (14)	C9—N2—C13	119.5 (5)
O14^i^—Ca1—O13^i^	92.24 (14)	C9—N2—Eu1	120.8 (3)
O14—Ca1—O13^i^	87.76 (14)	C13—N2—Eu1	119.7 (3)
O8^i^—Ca1—O13	91.70 (14)	C20—N3—C16	119.0 (5)
O8—Ca1—O13	88.30 (14)	C20—N3—Eu1	120.1 (3)
O14^i^—Ca1—O13	87.76 (14)	C16—N3—Eu1	120.8 (3)
O14—Ca1—O13	92.24 (14)	O2—C1—O1	125.8 (5)
O13^i^—Ca1—O13	180.0	O2—C1—C2	118.6 (5)
O8^i^—Ca1—H13A	92.6 (12)	O1—C1—C2	115.5 (5)
O8—Ca1—H13A	87.4 (12)	N1—C2—C3	122.4 (5)
O14^i^—Ca1—H13A	70.1 (6)	N1—C2—C1	114.5 (5)
O14—Ca1—H13A	109.9 (6)	C3—C2—C1	123.0 (5)
O13^i^—Ca1—H13A	162.2 (6)	C4—C3—C2	118.0 (6)
O13—Ca1—H13A	17.8 (6)	C4—C3—H3	121.0
O8^i^—Ca1—H14A	103.8 (11)	C2—C3—H3	121.0
O8—Ca1—H14A	76.2 (11)	C3—C4—C5	119.9 (6)
O14^i^—Ca1—H14A	162.7 (6)	C3—C4—H4	120.0
O14—Ca1—H14A	17.3 (6)	C5—C4—H4	120.0
O13^i^—Ca1—H14A	101.5 (10)	C6—C5—C4	118.6 (6)
O13—Ca1—H14A	78.5 (10)	C6—C5—H5	120.7
H13A—Ca1—H14A	95.5 (12)	C4—C5—H5	120.7
O6^ii^—Ca2—O6	78.34 (19)	N1—C6—C5	122.0 (6)
O6^ii^—Ca2—O2^iii^	113.44 (13)	N1—C6—C7	114.2 (5)
O6—Ca2—O2^iii^	141.21 (12)	C5—C6—C7	123.6 (5)
O6^ii^—Ca2—O2^iv^	141.21 (12)	O4—C7—O3	126.0 (5)
O6—Ca2—O2^iv^	113.44 (13)	O4—C7—C6	118.2 (5)
O2^iii^—Ca2—O2^iv^	81.20 (19)	O3—C7—C6	115.8 (5)
O6^ii^—Ca2—O15^ii^	78.83 (13)	O6—C8—O5	126.2 (5)
O6—Ca2—O15^ii^	72.14 (13)	O6—C8—C9	117.9 (5)
O2^iii^—Ca2—O15^ii^	144.61 (14)	O5—C8—C9	115.9 (5)
O2^iv^—Ca2—O15^ii^	71.03 (13)	N2—C9—C10	122.3 (5)
O6^ii^—Ca2—O15	72.14 (13)	N2—C9—C8	113.8 (5)
O6—Ca2—O15	78.83 (13)	C10—C9—C8	123.9 (5)
O2^iii^—Ca2—O15	71.03 (13)	C11—C10—C9	118.8 (5)
O2^iv^—Ca2—O15	144.61 (14)	C11—C10—H10	120.6
O15^ii^—Ca2—O15	142.3 (2)	C9—C10—H10	120.6
O6^ii^—Ca2—O16^ii^	74.59 (13)	C10—C11—C12	119.5 (5)
O6—Ca2—O16^ii^	145.52 (14)	C10—C11—H11	120.3
O2^iii^—Ca2—O16^ii^	70.74 (13)	C12—C11—H11	120.3
O2^iv^—Ca2—O16^ii^	77.45 (14)	C13—C12—C11	118.2 (5)
O15^ii^—Ca2—O16^ii^	81.92 (14)	C13—C12—H12	120.9
O15—Ca2—O16^ii^	111.92 (14)	C11—C12—H12	120.9
O6^ii^—Ca2—O16	145.52 (14)	N2—C13—C12	121.6 (5)
O6—Ca2—O16	74.59 (13)	N2—C13—C14	114.7 (5)
O2^iii^—Ca2—O16	77.45 (14)	C12—C13—C14	123.7 (5)
O2^iv^—Ca2—O16	70.74 (13)	O8—C14—O7	124.7 (5)
O15^ii^—Ca2—O16	111.91 (14)	O8—C14—C13	118.5 (5)
O15—Ca2—O16	81.92 (14)	O7—C14—C13	116.8 (5)
O16^ii^—Ca2—O16	137.8 (2)	O10—C15—O9	125.4 (5)
O6^ii^—Ca2—H15B	71.4 (11)	O10—C15—C16	118.1 (5)
O6—Ca2—H15B	61.0 (6)	O9—C15—C16	116.5 (5)
O2^iii^—Ca2—H15B	86.9 (6)	N3—C16—C17	121.9 (5)
O2^iv^—Ca2—H15B	147.2 (11)	N3—C16—C15	114.0 (5)
O15^ii^—Ca2—H15B	128.1 (6)	C17—C16—C15	124.1 (5)
O15—Ca2—H15B	17.9 (6)	C18—C17—C16	118.5 (6)
O16^ii^—Ca2—H15B	126.9 (9)	C18—C17—H17	120.7
O16—Ca2—H15B	76.9 (12)	C16—C17—H17	120.7
O12—Ca3—O12^v^	180.00 (9)	C17—C18—C19	119.3 (5)
O12—Ca3—O17^v^	93.91 (15)	C17—C18—H18	120.3
O12^v^—Ca3—O17^v^	86.09 (15)	C19—C18—H18	120.3
O12—Ca3—O17	86.09 (15)	C20—C19—C18	119.1 (6)
O12^v^—Ca3—O17	93.91 (15)	C20—C19—H19	120.5
O17^v^—Ca3—O17	180.0 (3)	C18—C19—H19	120.5
O12—Ca3—O18	90.53 (16)	N3—C20—C19	122.1 (5)
O12^v^—Ca3—O18	89.48 (16)	N3—C20—C21	114.6 (5)
O17^v^—Ca3—O18	95.84 (18)	C19—C20—C21	123.3 (5)
O17—Ca3—O18	84.16 (18)	O12—C21—O11	125.4 (5)
O12—Ca3—O18^v^	89.47 (16)	O12—C21—C20	118.5 (5)
O12^v^—Ca3—O18^v^	90.53 (16)	O11—C21—C20	116.1 (5)
O17^v^—Ca3—O18^v^	84.16 (18)	H19A—O19—H19B	109 (5)
O17—Ca3—O18^v^	95.84 (18)	H20A—O20—H20B	110 (5)
O18—Ca3—O18^v^	180.0	H21A—O21—H21B	111 (4)
O12—Ca3—H17B	72.8 (11)		
			
O5—Eu1—O1—C1	78.5 (4)	O11—Eu1—N3—C20	1.3 (4)
O7—Eu1—O1—C1	−152.5 (4)	O9—Eu1—N3—C20	−179.1 (4)
O3—Eu1—O1—C1	5.4 (5)	N2—Eu1—N3—C20	131.3 (4)
O11—Eu1—O1—C1	−72.0 (4)	N1—Eu1—N3—C20	−46.0 (4)
O9—Eu1—O1—C1	145.6 (4)	O5—Eu1—N3—C16	30.0 (5)
N2—Eu1—O1—C1	140.8 (4)	O7—Eu1—N3—C16	−98.5 (4)
N1—Eu1—O1—C1	3.2 (4)	O1—Eu1—N3—C16	−149.5 (4)
N3—Eu1—O1—C1	−101.8 (4)	O3—Eu1—N3—C16	82.3 (4)
O5—Eu1—O3—C7	−84.6 (4)	O11—Eu1—N3—C16	177.5 (4)
O7—Eu1—O3—C7	129.9 (4)	O9—Eu1—N3—C16	−2.9 (4)
O1—Eu1—O3—C7	−4.2 (5)	N2—Eu1—N3—C16	−52.5 (4)
O11—Eu1—O3—C7	68.7 (4)	N1—Eu1—N3—C16	130.2 (4)
O9—Eu1—O3—C7	−164.0 (4)	Ca2^vi^—O2—C1—O1	−8.5 (9)
N2—Eu1—O3—C7	−109.3 (4)	Ca2^vi^—O2—C1—C2	170.7 (3)
N1—Eu1—O3—C7	−2.1 (4)	Eu1—O1—C1—O2	179.0 (4)
N3—Eu1—O3—C7	131.4 (4)	Eu1—O1—C1—C2	−0.2 (6)
O7—Eu1—O5—C8	−0.5 (5)	C6—N1—C2—C3	2.3 (8)
O1—Eu1—O5—C8	72.5 (4)	Eu1—N1—C2—C3	−168.5 (4)
O3—Eu1—O5—C8	−159.0 (4)	C6—N1—C2—C1	179.4 (5)
O11—Eu1—O5—C8	140.8 (4)	Eu1—N1—C2—C1	8.6 (6)
O9—Eu1—O5—C8	−77.5 (4)	O2—C1—C2—N1	175.2 (5)
N2—Eu1—O5—C8	2.1 (4)	O1—C1—C2—N1	−5.5 (7)
N1—Eu1—O5—C8	135.1 (4)	O2—C1—C2—C3	−7.8 (8)
N3—Eu1—O5—C8	−107.2 (4)	O1—C1—C2—C3	171.5 (5)
O6^ii^—Ca2—O6—C8	66.7 (5)	N1—C2—C3—C4	−1.5 (9)
O2^iii^—Ca2—O6—C8	−46.3 (6)	C1—C2—C3—C4	−178.3 (5)
O2^iv^—Ca2—O6—C8	−152.2 (5)	C2—C3—C4—C5	−0.2 (9)
O15^ii^—Ca2—O6—C8	148.6 (5)	C3—C4—C5—C6	1.1 (10)
O15—Ca2—O6—C8	−7.1 (5)	C2—N1—C6—C5	−1.3 (8)
O16^ii^—Ca2—O6—C8	105.5 (5)	Eu1—N1—C6—C5	169.5 (4)
O16—Ca2—O6—C8	−91.7 (5)	C2—N1—C6—C7	−177.7 (5)
O5—Eu1—O7—C14	9.2 (4)	Eu1—N1—C6—C7	−7.0 (6)
O1—Eu1—O7—C14	−71.0 (4)	C4—C5—C6—N1	−0.4 (9)
O3—Eu1—O7—C14	145.0 (4)	C4—C5—C6—C7	175.7 (6)
O11—Eu1—O7—C14	−151.5 (4)	Eu1—O3—C7—O4	−179.7 (4)
O9—Eu1—O7—C14	81.2 (4)	Eu1—O3—C7—C6	−0.5 (7)
N2—Eu1—O7—C14	6.6 (4)	N1—C6—C7—O4	−175.9 (5)
N1—Eu1—O7—C14	−101.4 (4)	C5—C6—C7—O4	7.8 (9)
N3—Eu1—O7—C14	143.4 (4)	N1—C6—C7—O3	4.9 (7)
O14^i^—Ca1—O8—C14	−123.5 (8)	C5—C6—C7—O3	−171.4 (5)
O14—Ca1—O8—C14	56.5 (8)	Ca2—O6—C8—O5	−0.2 (9)
O13^i^—Ca1—O8—C14	144.1 (8)	Ca2—O6—C8—C9	178.4 (3)
O13—Ca1—O8—C14	−35.9 (8)	Eu1—O5—C8—O6	176.3 (4)
O5—Eu1—O9—C15	−157.9 (4)	Eu1—O5—C8—C9	−2.3 (6)
O7—Eu1—O9—C15	72.4 (4)	C13—N2—C9—C10	1.6 (8)
O1—Eu1—O9—C15	131.3 (4)	Eu1—N2—C9—C10	−176.9 (4)
O3—Eu1—O9—C15	−79.8 (4)	C13—N2—C9—C8	179.7 (4)
O11—Eu1—O9—C15	−1.2 (5)	Eu1—N2—C9—C8	1.2 (6)
N2—Eu1—O9—C15	136.1 (4)	O6—C8—C9—N2	−178.1 (4)
N1—Eu1—O9—C15	−104.8 (4)	O5—C8—C9—N2	0.6 (7)
N3—Eu1—O9—C15	−1.6 (4)	O6—C8—C9—C10	−0.1 (8)
O5—Eu1—O11—C21	133.3 (4)	O5—C8—C9—C10	178.7 (5)
O7—Eu1—O11—C21	−77.0 (4)	N2—C9—C10—C11	−1.0 (8)
O1—Eu1—O11—C21	−155.0 (4)	C8—C9—C10—C11	−178.9 (5)
O3—Eu1—O11—C21	75.8 (4)	C9—C10—C11—C12	−0.1 (8)
O9—Eu1—O11—C21	1.5 (5)	C10—C11—C12—C13	0.6 (8)
N2—Eu1—O11—C21	−106.3 (4)	C9—N2—C13—C12	−1.0 (7)
N1—Eu1—O11—C21	139.1 (4)	Eu1—N2—C13—C12	177.5 (4)
N3—Eu1—O11—C21	1.9 (4)	C9—N2—C13—C14	−179.4 (4)
O17^v^—Ca3—O12—C21	−129.5 (8)	Eu1—N2—C13—C14	−0.9 (6)
O17—Ca3—O12—C21	50.5 (8)	C11—C12—C13—N2	0.0 (8)
O18—Ca3—O12—C21	134.6 (8)	C11—C12—C13—C14	178.2 (5)
O18^v^—Ca3—O12—C21	−45.4 (8)	Ca1—O8—C14—O7	−107.4 (8)
O5—Eu1—N1—C6	85.5 (4)	Ca1—O8—C14—C13	73.3 (9)
O7—Eu1—N1—C6	−143.7 (4)	Eu1—O7—C14—O8	171.1 (4)
O1—Eu1—N1—C6	−176.9 (4)	Eu1—O7—C14—C13	−9.5 (6)
O3—Eu1—N1—C6	4.9 (4)	N2—C13—C14—O8	−174.2 (5)
O11—Eu1—N1—C6	−91.5 (4)	C12—C13—C14—O8	7.5 (8)
O9—Eu1—N1—C6	32.4 (5)	N2—C13—C14—O7	6.5 (7)
N2—Eu1—N1—C6	134.8 (4)	C12—C13—C14—O7	−171.9 (5)
N3—Eu1—N1—C6	−47.8 (4)	Eu1—O9—C15—O10	−173.7 (4)
O5—Eu1—N1—C2	−103.9 (4)	Eu1—O9—C15—C16	5.3 (7)
O7—Eu1—N1—C2	26.9 (5)	C20—N3—C16—C17	2.0 (8)
O1—Eu1—N1—C2	−6.3 (4)	Eu1—N3—C16—C17	−174.3 (4)
O3—Eu1—N1—C2	175.6 (4)	C20—N3—C16—C15	−177.5 (5)
O11—Eu1—N1—C2	79.2 (4)	Eu1—N3—C16—C15	6.3 (6)
O9—Eu1—N1—C2	−156.9 (3)	O10—C15—C16—N3	171.7 (5)
N2—Eu1—N1—C2	−54.5 (4)	O9—C15—C16—N3	−7.4 (7)
N3—Eu1—N1—C2	122.9 (4)	O10—C15—C16—C17	−7.7 (8)
O5—Eu1—N2—C9	−1.6 (3)	O9—C15—C16—C17	173.2 (5)
O7—Eu1—N2—C9	176.1 (4)	N3—C16—C17—C18	−1.8 (9)
O1—Eu1—N2—C9	−101.5 (4)	C15—C16—C17—C18	177.6 (5)
O3—Eu1—N2—C9	25.3 (4)	C16—C17—C18—C19	0.6 (9)
O11—Eu1—N2—C9	−151.8 (3)	C17—C18—C19—C20	0.4 (9)
O9—Eu1—N2—C9	81.1 (4)	C16—N3—C20—C19	−1.0 (8)
N1—Eu1—N2—C9	−57.2 (4)	Eu1—N3—C20—C19	175.3 (4)
N3—Eu1—N2—C9	125.5 (4)	C16—N3—C20—C21	−180.0 (5)
O5—Eu1—N2—C13	179.9 (4)	Eu1—N3—C20—C21	−3.7 (6)
O7—Eu1—N2—C13	−2.4 (3)	C18—C19—C20—N3	−0.2 (9)
O1—Eu1—N2—C13	80.0 (4)	C18—C19—C20—C21	178.7 (5)
O3—Eu1—N2—C13	−153.2 (3)	Ca3—O12—C21—O11	−98.7 (9)
O11—Eu1—N2—C13	29.7 (4)	Ca3—O12—C21—C20	81.6 (9)
O9—Eu1—N2—C13	−97.3 (4)	Eu1—O11—C21—O12	175.9 (4)
N1—Eu1—N2—C13	124.4 (4)	Eu1—O11—C21—C20	−4.4 (7)
N3—Eu1—N2—C13	−53.0 (4)	N3—C20—C21—O12	−175.2 (5)
O5—Eu1—N3—C20	−146.2 (4)	C19—C20—C21—O12	5.8 (8)
O7—Eu1—N3—C20	85.3 (4)	N3—C20—C21—O11	5.1 (7)
O1—Eu1—N3—C20	34.3 (5)	C19—C20—C21—O11	−173.9 (5)
O3—Eu1—N3—C20	−93.9 (4)		

Symmetry codes: (i) −*x*+1, −*y*, −*z*; (ii) −*x*+1, *y*, −*z*+1/2; (iii) *x*, *y*+1, *z*; (iv) −*x*+1, *y*+1, −*z*+1/2; (v) −*x*, −*y*, −*z*; (vi) *x*, *y*−1, *z*.

### Hydrogen-bond geometry (Å, º)

**Table:**

*D*—H···*A*	*D*—H	H···*A*	*D*···*A*	*D*—H···*A*
O13—H13*A*···O10^vii^	0.85 (1)	1.97 (2)	2.796 (6)	163 (5)
O13—H13*B*···O20^viii^	0.85 (1)	1.85 (1)	2.696 (6)	175 (6)
O14—H14*A*···O16^vi^	0.85 (1)	1.96 (2)	2.774 (6)	160 (5)
O14—H14*B*···O9^vi^	0.85 (1)	1.91 (2)	2.723 (5)	160 (5)
O15—H15*A*···O21^iii^	0.85 (1)	1.99 (1)	2.839 (6)	172 (6)
O15—H15*B*···O5	0.85 (1)	1.90 (1)	2.734 (5)	165 (5)
O16—H16*A*···O13^iii^	0.78 (6)	2.38 (7)	3.079 (6)	150 (6)
O16—H16*B*···O1^iii^	0.84 (6)	1.88 (7)	2.704 (6)	169 (6)
O17—H17*A*···O3^vi^	0.85 (1)	1.91 (1)	2.745 (6)	167 (5)
O17—H17*B*···O19^ix^	0.85 (1)	1.93 (3)	2.722 (7)	155 (6)
O18—H18*A*···O22′^x^	0.85 (1)	2.37 (6)	2.990 (15)	130 (6)
O18—H18*A*···O22^x^	0.85 (1)	2.16 (7)	2.657 (14)	117 (6)
O18—H18*B*···O4^vi^	0.85 (1)	1.98 (2)	2.772 (6)	155 (5)
O19—H19*A*···O24^xi^	0.86 (1)	2.31 (3)	3.12 (2)	159 (6)
O19—H19*B*···O7^xii^	0.85 (1)	2.07 (4)	2.828 (6)	148 (6)
O20—H20*A*···O15	0.85 (1)	2.04 (2)	2.862 (6)	161 (6)
O20—H20*B*···O23	0.85 (1)	2.03 (4)	2.758 (10)	143 (6)
O20—H20*B*···O22′^xiii^	0.85 (1)	2.32 (4)	3.042 (13)	143 (5)
O21—H21*A*···O11	0.85 (1)	1.94 (2)	2.774 (6)	167 (6)
O21—H21*B*···O17	0.85 (1)	2.42 (4)	3.113 (7)	138 (5)

Symmetry codes: (iii) *x*, *y*+1, *z*; (vi) *x*, *y*−1, *z*; (vii) −*x*+1, −*y*+1, −*z*; (viii) −*x*+1, *y*−1, −*z*+1/2; (ix) *x*, *y*, *z*−1; (x) −*x*, −*y*+1, −*z*+1; (xi) −*x*+1, −*y*+1, −*z*+1; (xii) *x*, *y*, *z*+1; (xiii) *x*, −*y*+2, *z*−1/2.
